# Supplementary material for: Photoelectrochemical Performance of Strontium Titanium Oxynitride Photo-Activated with Cobalt Phosphate Nanoparticles for Oxidation of Alkaline Water
Source: Nanomaterials (Basel). 2023 Mar 1;13(5):920. doi: 10.3390/nano13050920 (PMC10005293; doi:10.3390/nano13050920)
Supplement: Supplementary file 1 [file nanomaterials-13-00920-s001.zip › nanomaterials-2171938-supplementary.pdf]

nanomaterials

Supplementary Materials

Photoelectrochemical Performance of Strontium Titanium Oxynitride Photo-Activated with Cobalt Phosphate Nanoparticles for Oxidation of Alkaline Water

Mabrook S. Amer [1,2], Prabhakarn Arunachalam [1,*], Mohamed A. Ghanem [1,*], Abdullah M. Al-Mayouf [1,2] and Mark T. Weller [3]

1 Chemistry Department, College of Science, King Saud University, Riyadh 11451, Saudi Arabia
2 K.A.CAR Energy Research and Innovation Center at Riyadh, King Saud University, Riyadh 11451, Saudi Arabia
3 Chemistry Department, Cardiff University, Cardiff CF10 3AT, UK
* Correspondence: parunachalam@ksu.edu.sa (P.A.); mghanem@ksu.edu.sa (M.A.G.); Tel.: +966-114670405 (M.A.G.); Fax: +966-14675992 (M.A.G.)

1. EDX Elemental Analysis and XRD of Pristine SrTi(ON)$_{3-\delta}$ and CoPi/STON Films

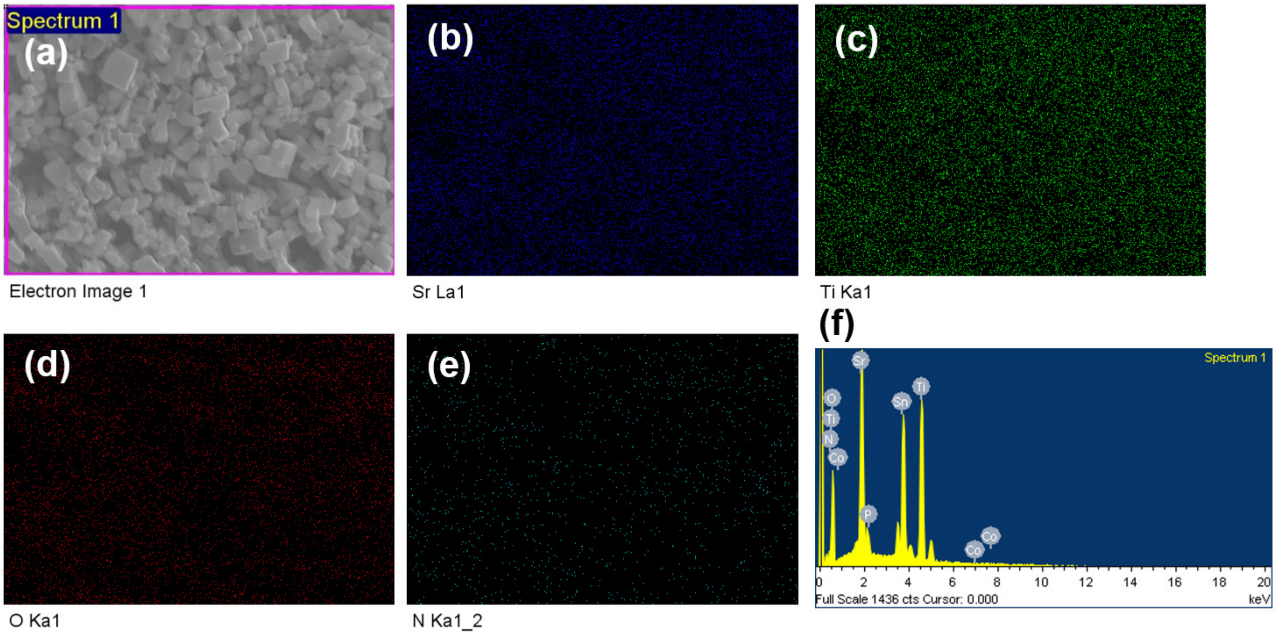

Figure S1. EDX elemental maps of pristine SrTi(ON)$_{3-\delta}$. SEM and the energy–dispersive X-ray EDX profile of Sr, Ti, O, and N elements of SrTi(O,N)$_{3-\delta}$ (**a**–**f**).

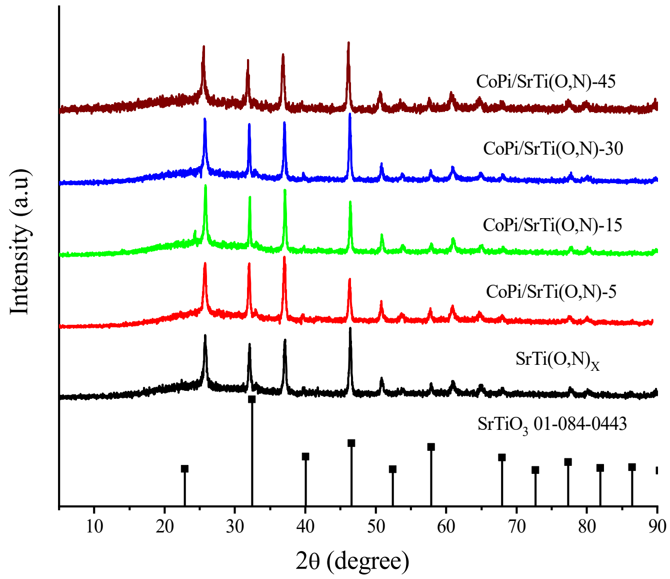

Figure S2. Normalized XRD patterns of SrTi(O,N), and CoPi/SrTi(O,N) photoanodes.

2-. The Electrochemical Active Surface Area and Double Layer Capacitance Determination

The ECSA was then assessed by evaluating the capacitive current related to double-layer charging from the sweep rate dependence of the cyclic voltammetry. The double-layer capacitance (C_{dl}) was assessed from the plot association between $\Delta J = (J_a - J_c)$ of and the sweep rate within the potential window of the capacitive region and ECSA was estimated according to the equation of ECSA = Cdl/Cs.

As shown in Figures S2c,d, the linear slope (C_{dl}) of CoPi/STON photoanodes is about eight times higher than that of the $SrTi(O,N)_{3-\delta}$ electrode which indicates a significant enhancement in the active site and ECSA upon the deposition of CoPi over the surface of $SrTi(O,N)_{3-\delta}$ electrode. Mostly, the specific capacitance (Cs) of 1 cm^2 flat surface area can be safely expected to have an average value of 40 $\mu F/cm^2$ [1].

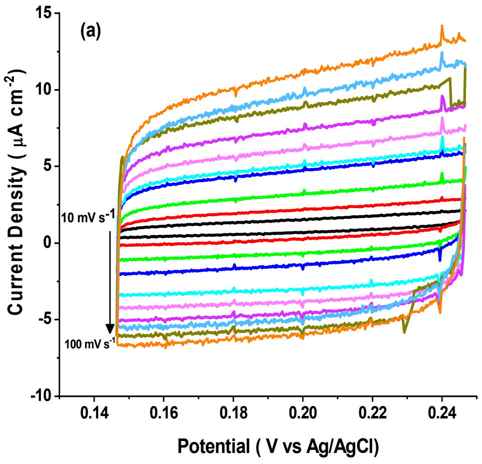

(**a**)

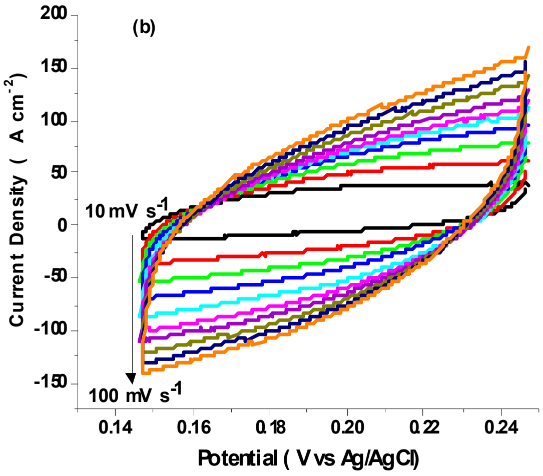

(**b**)

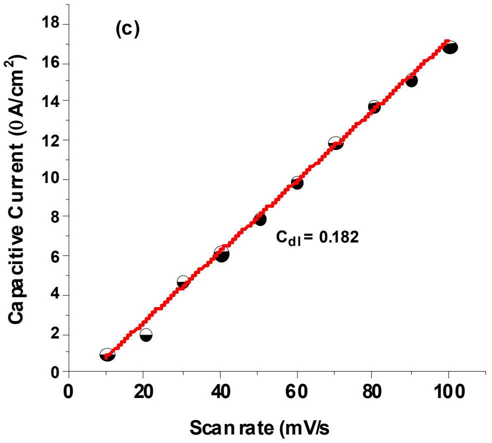

(**c**)

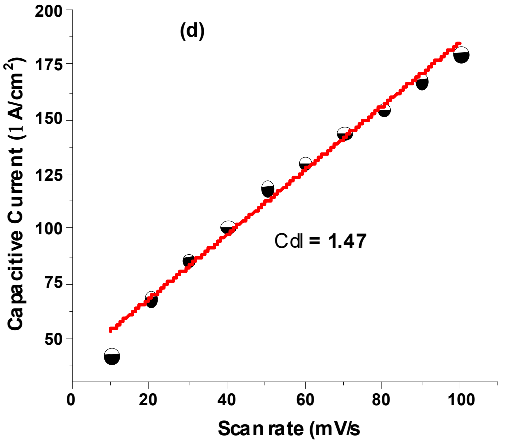

(**d**)

Figure S3. Cyclic voltammetry of (**a**) STON and (**b**) CoPi/STON electrodes carried out at various sweep scan rates in in 0.5 M NaOH (pH = 12.5) within the non-Faradaic potential region, charging current density differences of (**c**) STON (**d**) CoPi/STON electrodes plotted against scan rate.

3-. Films Characterization After Being Used in Electrolysis Process

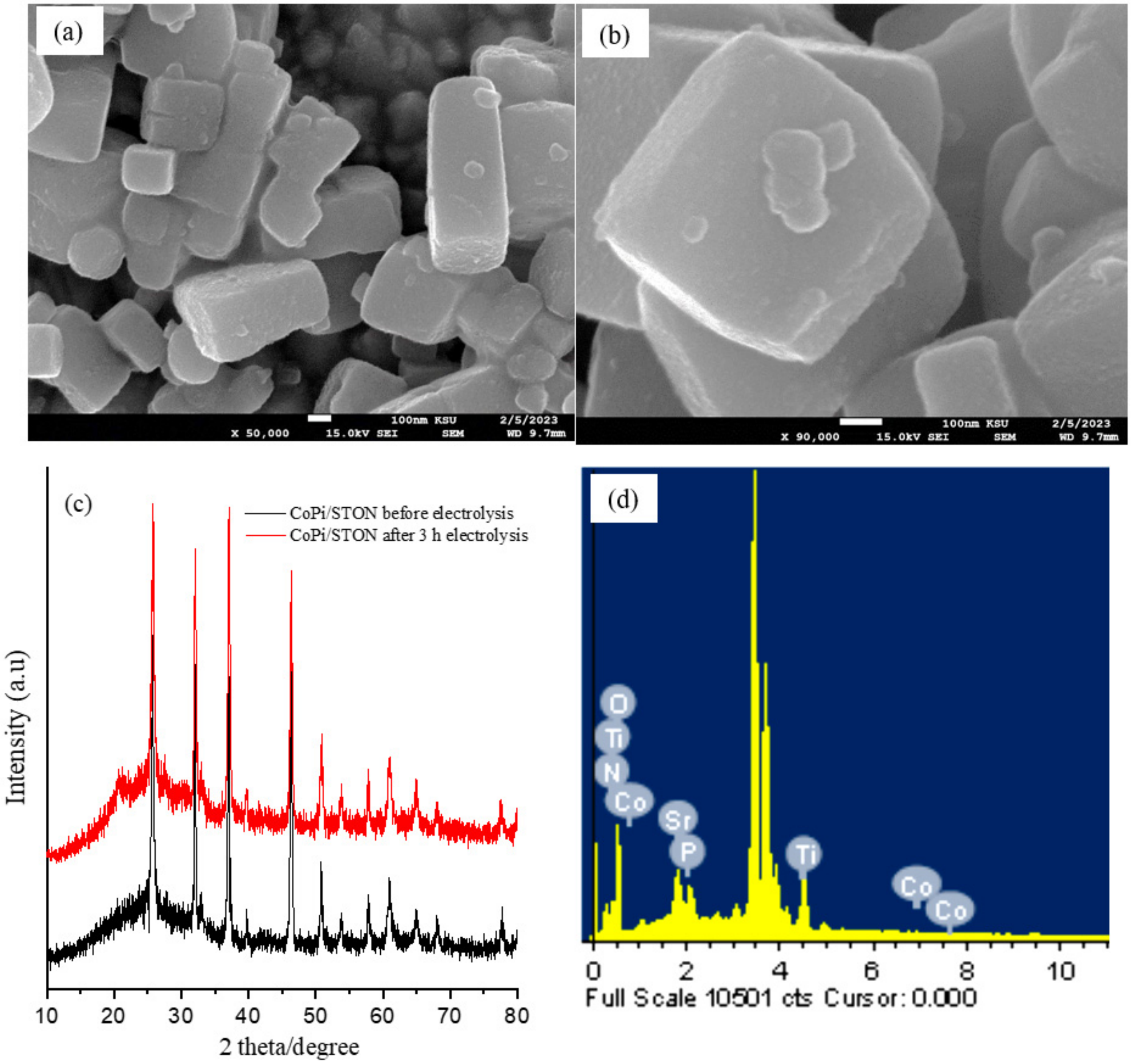

Figure S4. (**a**,**b**) The SEM images at different magnification of the CoPi/STON photoanode after been used in long-term stability test for 3 h and at an applied potential of 1.25 V vs. RHE and under light illumination in 0.5 M NaOH electrolyte, (**c**) comparison of XRD of CoPi/STON before and after stability test, and (**d**) the corresponding EDX elemental analysis of the CoPi/STON film after stability test.

References

[1] Liang, H.; Gandi, A. N.; Anjum, D. H.; Wang, X.; Schwingenschlögl, U.; Alshareef, Husam N. Plasma-Assisted Synthesis of NiCoP for Efficient Overall Water Splitting. *Nano Lett.* **2016**, *16*, 7718–7725.
